# Mapping the priority conservation areas for three endangered Cupressaceae plants under climate change in China

**DOI:** 10.3389/fpls.2024.1495442

**Published:** 2025-01-07

**Authors:** Lei Shen, Duanqiang Zhai, Xinyong Lu

**Affiliations:** ^1^ Zhejiang University-Shanghai Tunnel Engineering Co., Ltd. (ZJU-STEC), Urban Development and Planning Innovation Joint Research Center, Zhejiang University, Hangzhou, China; ^2^ China Eco-city Academy Co., Ltd., Tianjin, China; ^3^ College of Architecture and Urban Planning, Tongji University, Shanghai, China; ^4^ College of Economy and Trade, Zhongkai University of Agriculture and Engineering, Guangzhou, China

**Keywords:** endangered species, species distribution model, Marxan model, *Metasequoia glyptostroboides*, *Glyptostrobus pensilis*, *Thuja sutchuenensis*, global warming

## Abstract

The establishment of conservation areas is an important strategy for endangered species conservation. In this study, we investigated the distributions of suitable habitat areas for three level 1 endangered Cupressaceae plants (*Metasequoia glyptostroboides*, *Glyptostrobus pensilis*, and *Thuja sutchuenensis*) in China and used the Marxan model to delineate the priority conservation areas for each species. The results showed that *M. glyptostroboides* had the broadest suitable growing area under the current climate in China and is followed by *G. pensilis*, with an area of 91 × 10^4^ km^2^, and *T. sutchuenensis* had the smallest suitable habitat areas at only 7 × 10^4^ km^2^. Affected by climate change, the suitable ranges of these three Cupressaceae species moved largely northward at the middle and end of this century, with a latitudinal increase of 0.46–1.99°. *T. sutchuenensis* will face an extremely high extinction risk by the end of this century; 65.54% of its southern suitable habitat area will no longer be suitable for growth. Based on the effects of climate change, *M. glyptostroboides* priority conservation areas should be established in the Yangtze River Basin; *G. pensilis* priority conservation areas should be established in Guangdong, Guangxi, Fujian, and Jiangxi; and *T. sutchuenensis* protection districts should be established at the intersection of the northeastern part of Sichuan Province and the northern part of Chongqing. This study helps to clarify the impact of climate change on endangered species.

## 1 Introduction

Climate change profoundly impacts species morphology, physiology, and distribution (Root et al., 2003; Muluneh, 2021), and this poses enormous challenges to the conservation of biodiversity (Williams and Newbold, 2020; Kattel, 2022). Thomas et al. (2004) estimated that 15%–37% of species worldwide may become extinct due to climate warming. Regarding species that are already scarce, climate change will cause distribution fragmentation, a decrease in richness, and habitat loss (Ohlemüller et al., 2008; Hu and Jiang, 2011). Thus, there is an urgent need to increase attention and develop targeted conservation strategies to avoid species extinction.

The designation of conservation areas is a major strategy for the maximum protection of rare species (Haight and Hammill, 2020). This strategy has been studied in giant pandas (*Ailuropoda melanoleuca* David), snub-nosed monkeys (*Rhinopithecus* spp.), and black rhinoceros (*Diceros bicornis* Linnaeus), and great success has been achieved in the protection of endangered species (Odendaal-Holmes et al., 2014; Ellwanger et al., 2015; Guan et al., 2016). In contrast to endangered animals, which have received widespread attention, the protection status of wild plants is highly concerning. According to the International Union for Conservation of Nature Red List, 4,088 higher plant species are threatened in China, accounting for 10% of the total number of higher plants in China (Li et al., 2023). To address the protection of these endangered plant species, the Chinese government plans to integrate approximately 700 conservation areas, including national parks, by 2035 (Ye et al., 2023) and establish a national park covering approximately 10% of China’s land area and 80% of the conservation area. This will be known as a system of nature reserves for protected species.

The establishment of priority conservation areas at species-rich sites is currently the most widely used method globally, as it enhances regional biodiversity and is relatively straightforward to develop and implement (Ribeiro et al., 2017). However, a significant limitation of this approach is its inability to adequately protect endangered species that require special attention (Shrestha et al., 2019). In contrast, China’s conservation areas contain identified flagship species that inhabit specific regions or are of particular public concern, such as the Tropical Rainforest National Park in Hainan and the Dujiangyan Giant Panda Protection Research Center for the protection of giant pandas. While the establishment of these conservation areas has largely succeeded in protecting the target species, it has also been influenced by species conservation bias (Larsen et al., 2012; Shrestha et al., 2021). Consequently, the conservation priorities for endangered plants are generally low, and existing protected areas are insufficient to safeguard them.

The plant family Cupressaceae includes 32 genera in seven subfamilies and is the family with the most genera among gymnosperms (Liu et al., 2022). Owing to their origin in the high northern latitudes of the Cretaceous and Paleocene, Cupressaceae plants and many other Tertiary relict plants are known as “living fossils” in the plant kingdom. China’s “List of National Key Protected Wild Plants” includes 13 Cupressaceae plant species, among which five species have the highest degree of protection, level 1. Although the main risk faced by these five level 1 Cupressaceae is habitat loss, their current population sizes and reproductive statuses are different. *Metasequoia glyptostroboides* Hu & W. C. Cheng is one of the most successfully conserved plants worldwide and is now widely planted in the Northern Hemisphere (Li et al., 2005). *Glyptostrobus pensilis* (Staunton ex D. Don) K. Koch has also undergone artificial cultivation in China and is present at small scales in central Laos and southern Vietnam (Pueyo-Herrera et al., 2023). *Thuja sutchuenensis* Franch., *Cupressus gigantea* W. C. Cheng & L. K. Fu, and *Cupressus torulosa* D. Don are different from the abovementioned two species in that their population sizes are very small, artificial breeding techniques are not perfect, and they often maintain their populations through natural expansion (Xia et al., 2008; Gong et al., 2024; Zhang et al., 2024b).

Considering the differences among populations and the current situation, it is important to establish different conservation areas. Species distribution models (SDMs) based on niche theory are essential tools for predicting the potential suitable habitats of species (Marmion et al., 2009; Wen et al., 2024). Through SDMs, we can swiftly and accurately evaluate whether a specific area is suitable for designation as a conservation area for species. Among these models, ensemble models (EMs) utilize data from the extant distribution sites of the species and their associated environmental variables to explore its suitable environmental conditions for survival and to simulate potential suitable habitat within the research area. The Marxan model can repeatedly and randomly select a specified number of planning units, optimizing the least grid of protected areas to establish an effective conservation strategy (Foresta et al., 2016) and the rational delimitation of biologically prioritized protected areas from a biodiversity perspective, demonstrating strong applicability and operability. Through the combination of EM and Marxan (Tang et al., 2021), we can extract the ecological protection space of different species, which is highly important for the scientific planning of Cupressaceae plant reserves.

We used *M. glyptostroboides*, *G. pensilis*, and *T. sutchuenensis*, three endangered Cupressaceae plants, as the research subjects. This study aimed to (1) predict their potential distribution areas in China under current climatic conditions, (2) predict potential suitable habitats based on scenarios of global climate change over the 21st century and explore how their spatial configuration changes under different climatic scenarios, and (3) delineate the priority conservation areas using the Marxan model. Our study provides a new strategy for the conservation of endangered plants under climate change.

## 2 Materials and methods

### 2.1 Occurrence data

**Figure 1:**
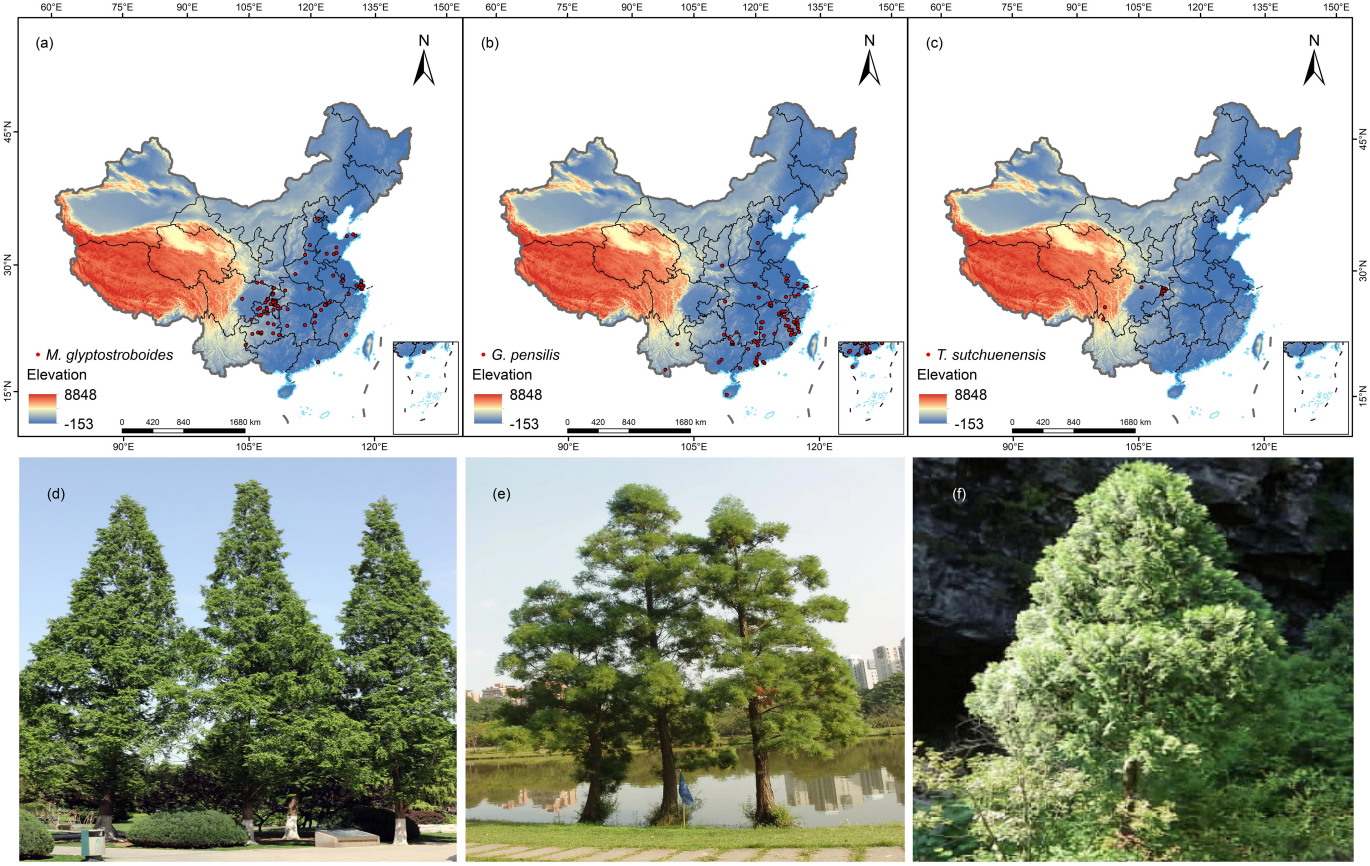
Distribution records and photos of three Cupressaceae plants: **(A–C)** distribution points of *Metasequoia glyptostroboides* Hu & W. C. Cheng, *Glyptostrobus pensilis* (Staunton ex D. Don) K. Koch, and *Thuja sutchuenensis* Franch. **(D–F)** Field photos of *M. glyptostroboides*, *G. pensilis*, and *T. sutchuenensis.*.

We collected the distribution data from the Chinese Virtual Herbarium (http://www.cvh.ac.cn/), Global Biodiversity Information Facility (https://www.gbif.org/), and China Knowledge Network (https://www.cnki.net/). The distribution data from 1980 to 2023 of the three Cupressaceae species and the latitude and longitude information were derived from relevant papers (Li et al., 2003; Li and Xia, 2004; Wang et al., 2008). The geographic area was set to mainland China. Since some point location data were recorded only in the sampling place names, the latitude and longitude were obtained through querying and positioning using the Baidu map pick-up coordinate system (http://api.map.baidu.com/lbsapi/getpoint/). We manually verified all the collected point information that did not include the type specimen to ensure its authenticity. The coordinates of the distributions of the three endangered Cupressaceae plants obtained after retrieval are shown in **Supplementary Table S1**. To avoid spatial autocorrelation between points, we used the R package spThin (Aiello-Lammens et al., 2015; Cheng et al., 2023; Zhang et al., 2024a) to delete duplicate points in the buffer area with a 4.5-km radius and retained 88 points for *M. glyptostroboides*, 79 points for *G. pensilis*, and 15 points for *T. sutchuenensis* (**Figure 1**).

### 2.2 Selection and processing of environmental variables

In this study, we selected 34 environmental variables from three categories: bioclimatic, soil, and topographic variables. The 19 bioclimatic variables at present (1970–2000), in the middle of the world (2050s: 2041–2060), and at the end of this century (2090s: 2081–2100) were downloaded from WorldClim version 2.1 (http://www.worldclim.org). The future climate conditions were the Beijing Climate Center-Climate System Model-Medium Resolution (BCC-CSM2-MR) in the Coupled Model Intercomparison Project Phase 6 (CMIP6) datasets, the pixel size of the data was 2.5 arc-minutes, and the level of development was intermediate development (SSP245). BCC-CSM2-MR has a higher resolution for both the atmosphere and the land surface and provides more detailed descriptions of the extreme temperature indices and trends across China (Wang et al., 2023). A total of 14 soil variables were obtained from the Food and Agriculture Organization of the United Nations World Soil Database (http://www.fao.org), and we used these data for both present and future projections. One elevation dataset was derived from a geospatial data cloud (http://www.gscloud.cn/). To avoid the impact of multicollinearity among the environmental variables on the model accuracy, we used the variance inflation factor (VIF) and Pearson correlations for screening. When the VIF is less than 5, there is no multicollinearity among factors; when the VIF is between 10 and 100, there is multicollinearity among factors; and when the VIF is greater than 100, there is serious interfactor multicollinearity (Dormann et al., 2013). We used the R package ENMeavl to screen for correlated environmental factors with sensitivities less than 0.7 and VIF values <5 (Muscarella et al., 2014). Each Cupressaceae species was individually screened, and the environmental factors associated with them were obtained separately (**Supplementary Table S2**).

### 2.3 Model construction and optimization

The 12 models included in Biomod2 were used to construct integrated models of species distributions. In this process, the “random” command was used to randomly generate pseudoexistence data points for use in model simulations. Through the “biomod – tuning” command, the model parameters were optimized, 75% of the sample data were selected as the training model, and the remaining 25% of the sample data were used to validate the model. The weights of the distribution data and the pseudodistribution data were set to be equal. This process was repeated 10 times to generate 120 model simulations. The accuracy of the prediction results was evaluated via the kappa coefficient, AUC, and TSS (Allouche et al., 2006). The weights for the model combinations were determined on the basis of the AUC and TSS values of each model, and the single models that were used to construct the ensemble models were determined with fixed cutoffs of TSS >0.7 and AUC >0.8 (Cheng et al., 2023). The higher the average AUC and TSS values were after multiple runs, the greater the weight assigned to the corresponding single model was when it was incorporated into the ensemble model. Single models with accuracies that met the selected standard were integrated into an ensemble model via a weighted average approach.

### 2.4 Changes in the spatial pattern of the suitable distribution area

The cutoff values of suitable/not suitable (0/1) were calculated by using the binary.meth function in Biomod2, and areas lower than the cutoff value were considered areas with unsuitable habitat. The areas above the cutoff value were divided into three equal parts, corresponding to areas with low, medium, and high suitability (Wen et al., 2022). The division results were loaded into ArcGIS v10.4.1 for visual presentation. The differences in suitable habitat areas for the studied species in different periods were compared via the range size function to obtain a map of the changes in the species spatial distribution patterns under future climate change scenarios. The R packages “SDMTool” and “geosphere” were used to calculate the positions of the centroids and the migration distances of the current and future suitable zones of the species, respectively, and the changes in centroid locations were obtained to represent the migration directions of the spatial distributions of the suitable zones for the three species (Gao et al., 2023).

### 2.5 Priority conservation areas

The Marxan model was used to identify the areas in the protection system with the lowest cost and the highest suitability index as priority protection areas (Xie et al., 2022). The priority protection area was determined according to the following steps: (1) planning cell and cost setting: The current distribution range obtained by Biomod2 was used as the research subject, and a 1-hm^2^ grid was used as the research unit. Each unit was assigned a unique ID value, and the area of each cell was set as the protection cost. The species distribution matrix was constructed, and the habitat area was calculated via zonal statistics, as shown in ArcGIS; (2) conservation target of the species: Since the three cypresses are the highest-grade protected plants, we chose the highest ratio of total habitat area (the probability of the species existence value greater than the critical value is 90%) and set the protection target (Foresta et al., 2016); and (3) Marxan run: ArcGIS was used to add the plug-in, ArcMarxan2.pyt, to generate the unit boundary lengths. The number of software iterations was set to 1,000,000 (Cheng et al., 2023), and the species penalty factor and boundary length modifier were constantly modified for optimization while the other parameters remained unchanged (Mou et al., 2021). After the iterative calculation of the location selection, the spatial compactness of the adjusted result units was controlled by the boundary length modifier (BLM) of the model. If it was too dense, some planning units with low protection effects may have been selected; as a result, the protected area distribution was too discrete (Zhang et al., 2011). Then, the cost of the results and the relationship between the total boundary length and the total area could be analyzed via BLM modification. The larger the BLM value was, the more crucial the boundary cost and the smaller the fragmentation degree. The final model used a model boundary correction value of 100.

## 3 Results

### 3.1 Model accuracy evaluation

**Figure 2:**
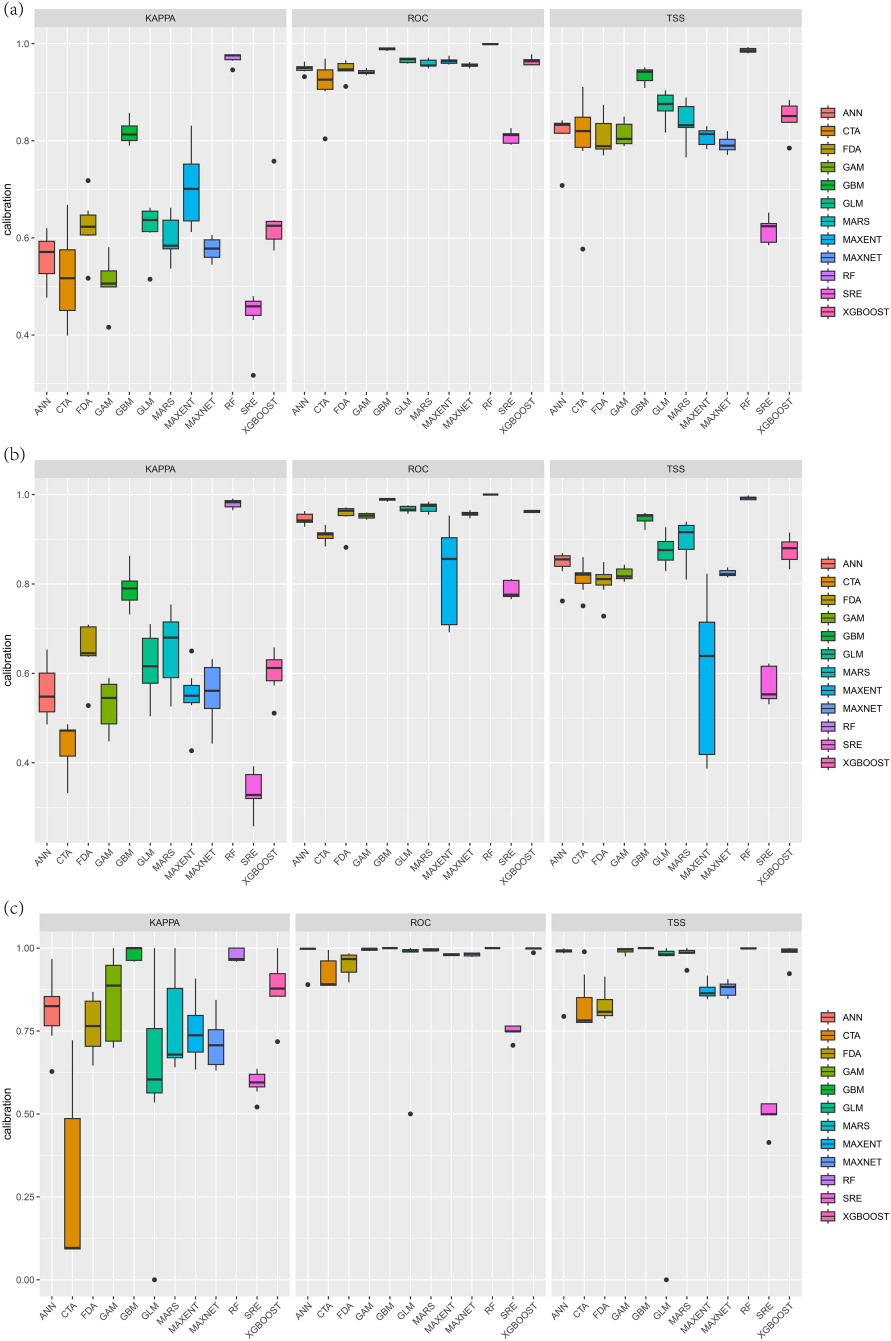
Comparison of the AUCs, kappa coefficients, and TSSs of the 12 models: **(A)** *Metasequoia glyptostroboides* Hu & W. C. Cheng, **(B)** *Glyptostrobus pensilis* (Staunton ex D. Don) K. Koch, and **(C)** *Thuja sutchuenensis* Franch.

The accuracy results (**Figure 2**) revealed that the generalized boosted regression model (GBM) and random forest (RF) model were the optimal models for predicting the potential adaptive regions of the three Cupressaceae species. With respect to the three evaluation indicators, they all had high accuracy and good stability; the worst performing models were the flexible discriminant analysis (FDA) and surface range envelope (SRE) models, which failed the model accuracy test. The performances of several other models were between those of the abovementioned models. From the 120 model results, an excellent model with a true skill statistic (TSS) value greater than 0.7 was screened to construct a combination model. The kappa coefficients for the final three Cupressaceae plant ensemble models were 0.73, 0.75, and 0.86; the TSS values were 0.85, 0.87, and 0.99; and the AUCs of the receiver operating characteristic (ROC) curves were 0.97, 0.97, and 0.99, respectively, which were excellent.

### 3.2 Current period potential distribution area

**Figure 3:**
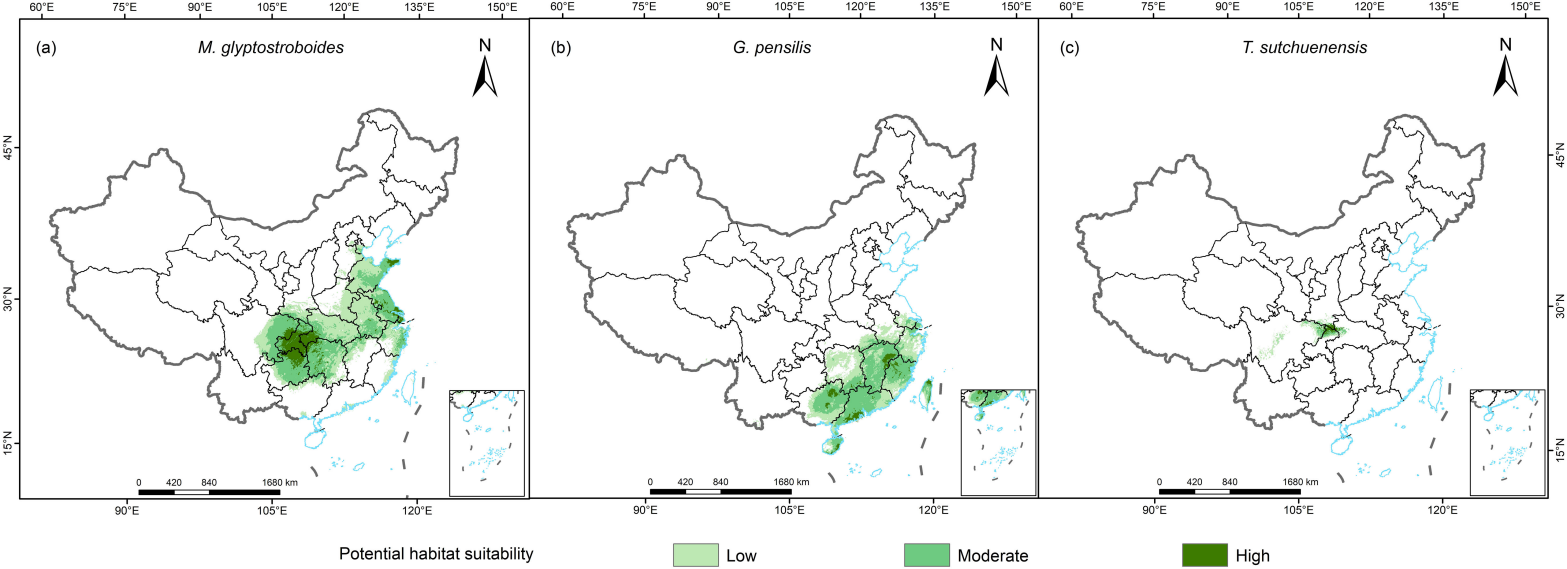
Current habitat suitability areas for the three Cupressaceae plants. **(A)** *Metasequoia glyptostroboides* Hu & W. C. Cheng, **(B)** *Glyptostrobus pensilis* (Staunton ex D. Don) K. Koch, and **(C)** *Thuja sutchuenensis* Franch.

The potential distribution areas of the three Cupressaceae plants under the current climate scenario are shown in **Figure 3**. The total area of suitable habitat for *M. glyptostroboides* was 143.07 × 10^4^ km^2^, in which the high-suitability zone consisted of 16.96 × 10^4^ km^2^, and the medium- and low-suitability zones consisted of 126.11 × 10^4^ km^2^ (**Table 1**). The areas with suitable habitat for *M. glyptostroboides* were mostly located in East, Central, and Southwest China, and Chongqing City was the area with the highest suitability for *M. glyptostroboides*. The total area with a suitable habitat for *G. pensilis* was 91.22 × 10^4^ km^2^, of which the area of highly suitable habitat was 5.24 × 10^4^ km^2^, which accounted for only 5.7% of the total area of suitable habitat. The areas with suitable habitat were mainly concentrated on the southeastern coast of China. The area at the junction of Sichuan, Chongqing, and Hubei provinces was the only area that was suitable for the growth of *Thuja sutchuenensis*, with a total suitable habitat area of 7.36 × 10^4^ km^2^.

**Table 1:** Areas of suitable habitat for the three Cupressaceae plants under the current scenarios.

Species	Low suitable habitat area (×10^4^ km^2^)	Moderate suitable habitat area (×10^4^ km^2^)	High suitable habitat area (×10^4^ km^2^)	Total suitable habitat area (×10^4^ km^2^)
*Metasequoia glyptostroboides* Hu & W. C. Cheng	67.8	58.31	16.96	143.07
*Glyptostrobus pensilis* (Staunton ex D. Don) K. Koch	40.55	45.43	5.24	91.22
*Thuja sutchuenensis* Franch.	3.85	2.18	1.31	7.36

### 3.3 Predicted future potential distribution areas

**Figure 4:**
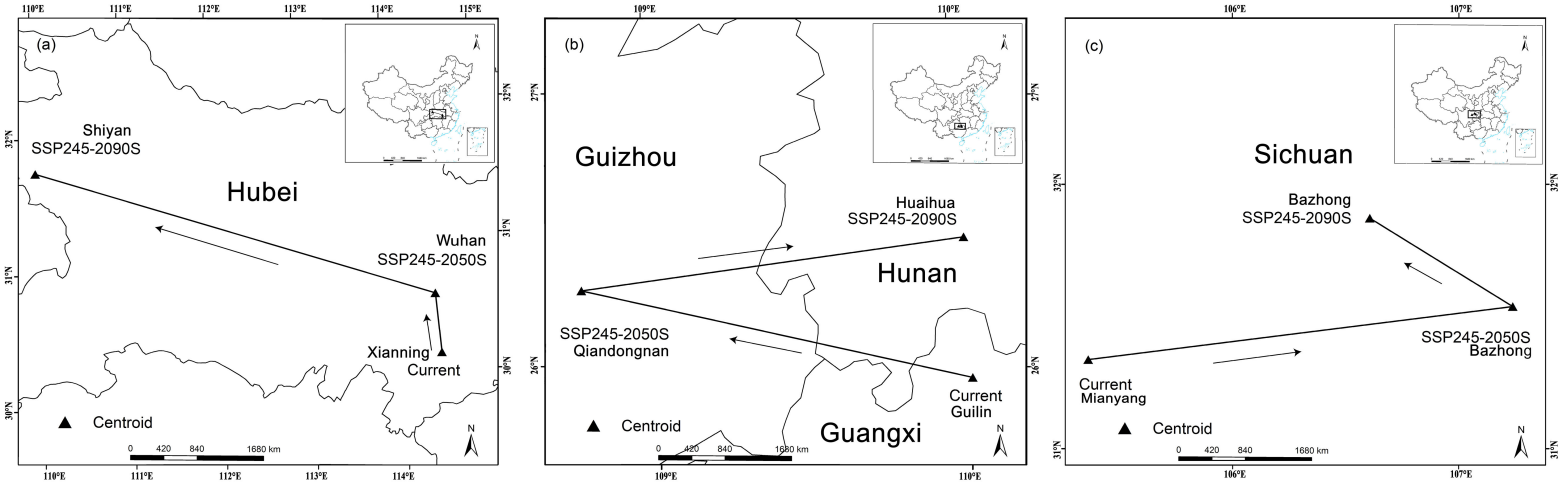
Three Cupressaceae plant niches under SSP245: **(A)** *Metasequoia glyptostroboides* Hu & W. C. Cheng, **(B)** *Glyptostrobus pensilis* (Staunton ex D. Don) K. Koch, and **(C)** *Thuja sutchuenensis* Franch.

The potential distribution areas of the three Cupressaceae plants all showed clear shifts to high latitudes (**Figure 4**). The centroid of the suitable habitat area for *M. glyptostroboides* was located in Xianning City (114.31° E, 29.84° N), the centroid for *G. pensilis* was located in Guilin City (110.00° E, 25.96° N), and the centroid for *T. sutchuenensis* was located in Mianyang City (105.38° E, 31.34° N). In 2050, *T. sutchuenensis* had the farthest northward migration distance of 17.4 km, and the migration distance of the other two Cupressaceae plants also reached 6 km or more. At the end of this century, the centroids of *M. glyptostroboides*, *G. pensilis*, and *T. sutchuenensis* were in Shiyan City, Hubei Province (109.79° E, 31.83° N), Huaihua City, Hunan Province (109.99° E, 26.42° N), and Bazhong City, Sichuan Province (106.60° E, 31.85° N). Compared with the current latitudes, the latitudes will increase by more than 0.4°.

**Figure 5:**
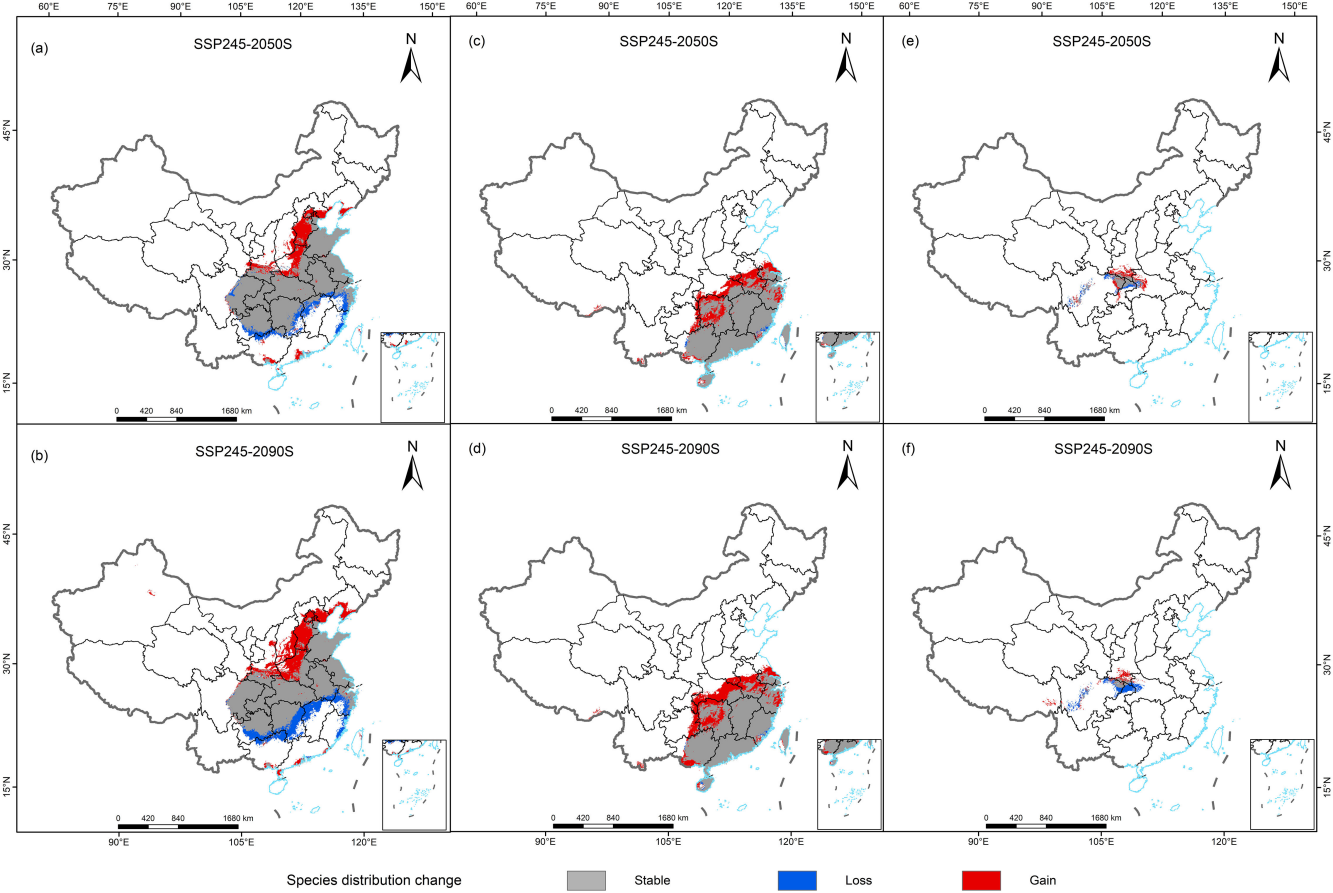
Spatial changes in the geographic distributions of three Cupressaceae plants in 2050s and 2090s: **(A, B)** *Metasequoia glyptostroboides* Hu & W. C. Cheng, **(C, D)** *Glyptostrobus pensilis* (Staunton ex D. Don) K. Koch, and **(E, F)** *Thuja sutchuenensis* Franch.

The simulation results revealed that the total distribution areas of suitable habitat for the three Cupressaceae plants also changed greatly under the future climate change scenarios (**Table 2**). In the middle of this century, the areas suitable for the three Cupressaceae plants all increased significantly, with the proportions of *G. pensilis* and *T. sutchuenensis* both reaching more than 25%. By the end of this century, the changes in the suitable habitat areas for *T. sutchuenensis* will be far from ideal, and many suitable habitat areas in the southern region will experience losses. The prediction results for *G. pensilis* were relatively good and continued to have the same increasing trend as that in the 2050s, and the areas with increases were mainly located along the Yangtze River. The suitable habitat areas for *M. glyptostroboides* were the least affected by climate change, maintaining the lowest increase or decrease in the two periods. These results were also confirmed by the changes in the red and blue areas in **Figure 5**.

**Table 2:** Changes in the distribution areas of three Cupressaceae plants in different periods.

Period	Species	Habitat area (×10^4^ km^2^)	Loss (×10^4^ km^2^)	Stable (×10^4^ km^2^)	Gain (×10^4^ km^2^)	Species range change (%)	Percentage loss (%)	Percentage gain (%)
2050s	*Metasequoia glyptostroboides* Hu & W. C. Cheng	153.75	14.34	128.74	25.01	7.46	10.02	17.48
	*Glyptostrobus pensilis* (Staunton ex D. Don) K. Koch	115.99	0.78	90.44	25.54	27.15	0.86	28.00
	*Thuja sutchuenensis* Franch.	9.33	2.11	5.25	4.08	26.77	28.66	55.44
2090s	*Metasequoia glyptostroboides* Hu & W. C. Cheng	157.46	22.57	120.50	36.96	10.06	15.78	25.83
	*Glyptostrobus pensilis* (Staunton ex D. Don) K. Koch	123.09	0.55	90.68	32.42	34.94	0.60	35.53
	*Thuja sutchuenensis* Franch.	4.85	4.82	2.54	2.31	-34.11	65.54	31.42

### 3.4 Priority conservation areas

**Figure 6:**
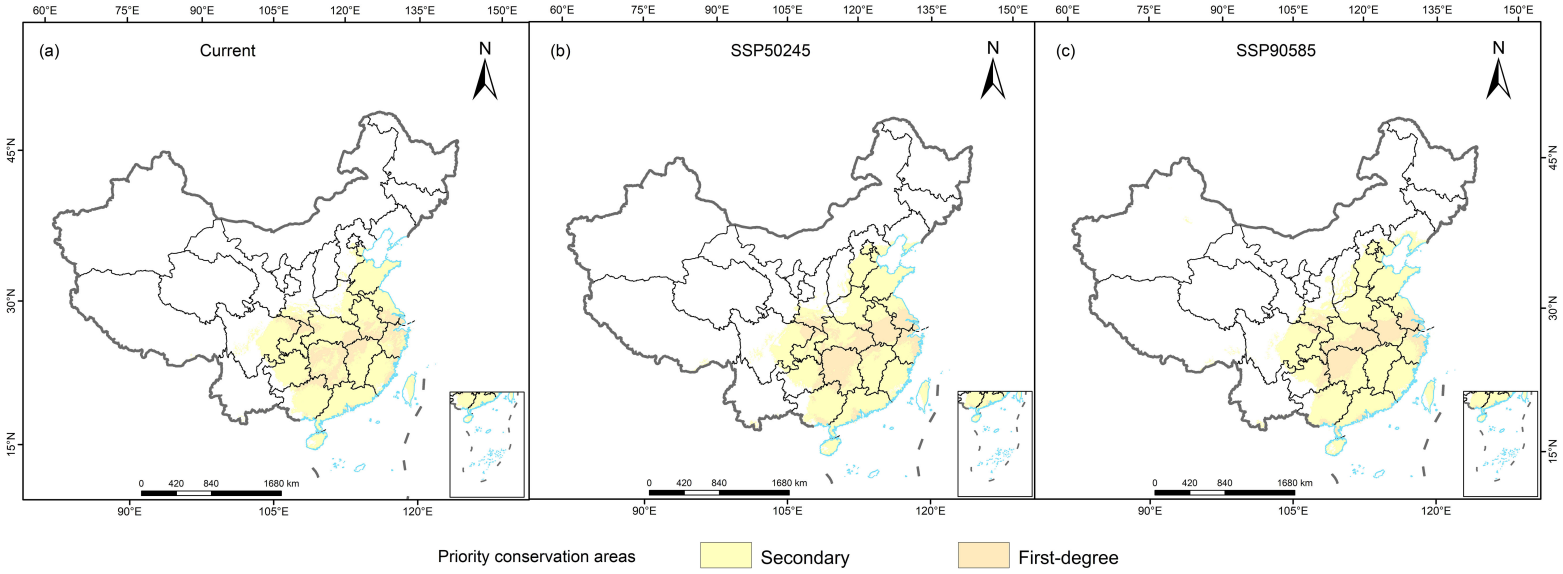
Priority conservation area plan for three Cupressaceae species: **(A)** current, **(B)** mid-century, and **(C)** end of the century.

With the goal of including 90% of the areas with suitable habitat, the Marxan model was used to identify priority conservation areas (**Figure 6**). The priority conservation areas for the three Cupressaceae species were 92.04 × 10^4^ km^2^ (*M. glyptostroboides*), 76.73 × 10^4^ km^2^ (*G. pensilis*), and 6.47 × 10^4^ km^2^ (*T. sutchuenensis*), which accounted for approximately 9.59%, 7.99%, and 0.74% of the total land area of China, respectively (**Supplementary Figure S1**). Except for *M. glyptostroboides*, the priority conservation areas in the 2050s were expected to decrease, and the future increases and decreases in the other conservation areas were highly consistent with the changes in the suitable habitat areas. Based on the current distribution and future climate change, the *M. glyptostroboides* protection areas were in Hubei, Sichuan, and Chongqing as well as in the central coastal areas of Shanghai, Jiangsu, and Anhui. The priority conservation areas of *G. pensilis* were in Guangdong, Guangxi, Fujian, and Jiangxi. The *T. sutchuenensis* reserve was at the junction of Dazhou City and the northern part of Chongqing City in northeastern Sichuan Province.

## 4 Discussion

### 4.1 Conservation status of the three Cupressaceae plants in the current period and recommendations for future planning of protected areas

Overall, it appears that less than 10% of the conservation priority areas for the three Cupressaceae species in China are protected by nature reserves; for *T. sutchuenensis*, this figure is less than 1%. By comparing the conservation unit preferences of the three Cupressaceae plants with the existing conservation patterns in China, the results of the conservation gap analysis indicate that significant gaps remain in the conservation efforts within the study area. Cupressaceae plants are native tree species with significant comprehensive utilization value and development potential in China, particularly in the commercial wood sector, where they are highly valuable (Polman et al., 1999). However, due to the unique genetic characteristics of Cupressaceae plants and their long-term adaptation to environmental conditions throughout the phylogenetic process, their ecological amplitude has narrowed, and their ability to reproduce independently has diminished. This decline is a significant factor contributing to the vulnerability of rare and endangered species in the current context. Therefore, optimizing and adjusting nature reserves based on the findings of the conservation priority areas identified in this study can serve as a scientific reference for the establishment of protected area networks for Cupressaceae plants in the future. This approach will provide a solid foundation for conducting conservation efforts for endangered species following thorough feasibility studies.

### 4.2 The artificial breeding technology for Cupressaceae plants still has limitations

In recent decades, great progress has been made in artificial propagation technology for endangered plants. For the three Cupressaceae plants in this study, artificial sowing, cutting, and tissue culture of field populations have been completed successively. In addition to *T. sutchuenensis*, the other two plants have also been grafted (Deng et al., 2020). However, compared with the wild population, artificial breeding has many problems—for example, the artificially bred *M. glyptostroboides* all come from the mother plant in Lichuan City, Hubei Province, China, which cannot be renewed naturally, and the population is degraded (Xiong et al., 2020). Moreover, *G. pensilis* has a high seed malformation rate (Dörken and Rudall, 2018), and its survival rates after sowing and cutting are very low. Moreover, *T. sutchuenensis* has low genetic diversity (Tao et al., 2024), its seed setting is very low, and severe abortion occurs. In view of these problems, it is important to continue to protect local wild populations.

### 4.3 Impact of climate warming on the geographical distribution of Cupressaceae plants

In the future, the potential distribution of *G. pensilis* and *T. sutchuenensis* in China differs significantly from their current distribution. This suggests that future climate warming will have a notable impact on the geographical distribution of *G. pensilis* and *T. sutchuenensis*. The response of three Cupressaceae plants to climate change in 2090s was significantly greater than that in 2050s, which indicated that climatic factors predominantly influence the geographical distribution of Cupressaceae plants. The most significant change observed in *T. sutchuenensis* among the three Cupressaceae species indicates that a smaller distribution area correlates with a greater impact from climate change.

The impact of climate warming on the potential geographic distribution of the species is primarily evident in the shift of their distribution areas toward higher latitudes or altitudes as well as in the expansion and contraction of these areas (Mason et al., 2015). Our results showed that the new areas suitable for the three Cupressaceae plants in the future are all in the northern part of the current major distribution areas, which is highly consistent with the general consensus in the scientific community—that is, a rise in temperature will cause the species to move to cooler places. However, practically speaking, whether plants can complete such large-scale northward movement is a problem that needs to be considered—for example, *G. pensilis* has a moderate level of protection. In the 1950s, *G. pensilis* was found everywhere in the Pearl River Delta region of China, but there were only 14 individuals in Guangzhou City (Chen et al., 2016). The training of *G. pensilis* has increased in all areas of suitable growth areas, but in most areas, *G. pensilis* is distributed only sporadically (Ye et al., 2022) and cannot form a complete forest. Coupled with the constraints of the inability to grow continuously, if effective protection measures are not adopted, *G. pensilis* will face extinction in the next 100 years (Thomas and LePage, 2011).

### 4.4 Limitations of the models’ predictions in future periods and further outlooks

Although we adopted a relatively conservative modeling method, we should note that, as a modeling study, this study is inevitably limited. On the one hand, only the bioclimatic factors from the corresponding period were utilized to predict potential suitable habitats under various future climate scenarios. Since obtaining future soil and topographic data is challenging, the default parameters remain unchanged from the present. However, in reality, soil properties, such as salinity saturation, and topographic factors, such as slope, also influence potential geographic distribution. On the other hand, the distribution and extinction risk of a species are affected by the comprehensive interplay of species traits, local geological conditions, and human activities (Sandler, 2014). Therefore, when establishing a protected area, it is crucial to consider whether the area is a densely populated urban environment or if other specific circumstances render it unsuitable for the establishment of a nature reserve. Nevertheless, the results of this study provide valuable theoretical support for the development of reasonable adaptation strategies for rare native tree species in response to climate change. They are significant in guiding the conservation of germplasm resources, future planting plans, and the sustainable development and utilization of Cupressaceae plants.

## 5 Conclusions

In summary, the EM successfully simulated the distributions of areas with suitable habitat for *M. glyptostroboides*, *G. pensilis*, and *T. sutchuenensis*, three level 1 endangered plants in China. Under the future climate change scenario, the areas with suitable habitat for these three Cupressaceae plants all experienced large high-latitude shifts. The suitable habitat area for *T. sutchuenensis* decreased by 65.54% by the end of this century, indicating the highest risk of extinction. The Marxan model revealed that the best conservation areas for *M. glyptostroboides* are in Hubei, Sichuan, Chongqing, Jiangsu, and Anhui; the priority conservation areas for *G. pensilis* are in Guangdong, Guangxi, Fujian, and Jiangxi; and the priority conservation area for *T. sutchuenensis* is in Sichuan Province (specifically, at the intersection of Dazhou City and northern Chongqing City in Northeast China). This study helps strengthen the protection of endangered plants and identify the priority conservation areas.

## Data Availability

The original contributions presented in the study are included in the article/**Supplementary Material**. Further inquiries can be directed to the corresponding author.
