# Supplementary material for: Mapping the priority conservation areas for three endangered Cupressaceae plants under climate change in China
Source: Front Plant Sci. 2025 Jan 7;15:1495442. doi: 10.3389/fpls.2024.1495442 (PMC11745887; doi:10.3389/fpls.2024.1495442)
Supplement: Supplementary file 1 [file DataSheet1.docx]

Supplementary Material

Table S1: Distribution records of *Metasequoia glyptostroboides* Hu & W. C. Cheng, *Glyptostrobus pensilis* (Staunton ex D. Don) K. Koch, and *Thuja sutchuenensis* Franch*.*

| Number | Species | Longitude | Latitude | Data sources |
| --- | --- | --- | --- | --- |
| 1 | *Metasequoia glyptostroboides* Hu & W. C. Cheng | 110.3 | 31.6 | GBIF.org (06 March 2024) GBIF Occurrence Download https://doi.org/10.15468/dl.bkdaje |
| 2 | *Metasequoia glyptostroboides* Hu & W. C. Cheng | 118.9 | 32.2 | GBIF.org (06 March 2024) GBIF Occurrence Download https://doi.org/10.15468/dl.bkdaje |
| 3 | *Metasequoia glyptostroboides* Hu & W. C. Cheng | 107.5 | 29.6 | GBIF.org (06 March 2024) GBIF Occurrence Download https://doi.org/10.15468/dl.bkdaje |
| 4 | *Metasequoia glyptostroboides* Hu & W. C. Cheng | 115.2 | 28.9 | GBIF.org (06 March 2024) GBIF Occurrence Download https://doi.org/10.15468/dl.bkdaje |
| 5 | *Metasequoia glyptostroboides* Hu & W. C. Cheng | 121.4 | 31.4 | GBIF.org (06 March 2024) GBIF Occurrence Download https://doi.org/10.15468/dl.bkdaje |
| 6 | *Metasequoia glyptostroboides* Hu & W. C. Cheng | 121.5 | 31.7 | GBIF.org (06 March 2024) GBIF Occurrence Download https://doi.org/10.15468/dl.bkdaje |
| 7 | *Metasequoia glyptostroboides* Hu & W. C. Cheng | 106.9 | 32.7 | GBIF.org (06 March 2024) GBIF Occurrence Download https://doi.org/10.15468/dl.bkdaje |
| 8 | *Metasequoia glyptostroboides* Hu & W. C. Cheng | 104.0 | 30.7 | GBIF.org (06 March 2024) GBIF Occurrence Download https://doi.org/10.15468/dl.bkdaje |
| 9 | *Metasequoia glyptostroboides* Hu & W. C. Cheng | 120.7 | 31.3 | GBIF.org (06 March 2024) GBIF Occurrence Download https://doi.org/10.15468/dl.bkdaje |
| 10 | *Metasequoia glyptostroboides* Hu & W. C. Cheng | 108.4 | 30.5 | GBIF.org (06 March 2024) GBIF Occurrence Download https://doi.org/10.15468/dl.bkdaje |
| 11 | *Metasequoia glyptostroboides* Hu & W. C. Cheng | 110.5 | 29.3 | GBIF.org (06 March 2024) GBIF Occurrence Download https://doi.org/10.15468/dl.bkdaje |
| 12 | *Metasequoia glyptostroboides* Hu & W. C. Cheng | 108.5 | 31.3 | GBIF.org (06 March 2024) GBIF Occurrence Download https://doi.org/10.15468/dl.bkdaje |
| 13 | *Metasequoia glyptostroboides* Hu & W. C. Cheng | 119.4 | 30.4 | GBIF.org (06 March 2024) GBIF Occurrence Download https://doi.org/10.15468/dl.bkdaje |
| 14 | *Metasequoia glyptostroboides* Hu & W. C. Cheng | 109.104543 | 26.202674 | Chinese Virtual Herbarium |
| 15 | *Metasequoia glyptostroboides* Hu & W. C. Cheng | 109.104543 | 26.202674 | Chinese Virtual Herbarium |
| 16 | *Metasequoia glyptostroboides* Hu & W. C. Cheng | 116.9626889 | 35.59783056 | Chinese Virtual Herbarium |
| 17 | *Metasequoia glyptostroboides* Hu & W. C. Cheng | 106.24319 | 26.58335 | Chinese Virtual Herbarium |
| 18 | *Metasequoia glyptostroboides* Hu & W. C. Cheng | 108.31155 | 26.44228 | Chinese Virtual Herbarium |
| 19 | *Metasequoia glyptostroboides* Hu & W. C. Cheng | 106.5524 | 26.504 | Chinese Virtual Herbarium |
| 20 | *Metasequoia glyptostroboides* Hu & W. C. Cheng | 104.53023 | 25.04234 | Chinese Virtual Herbarium |
| 21 | *Metasequoia glyptostroboides* Hu & W. C. Cheng | 106.242871 | 29.413392 | Chinese Virtual Herbarium |
| 22 | *Metasequoia glyptostroboides* Hu & W. C. Cheng | 116.976982 | 35.603611 | Chinese Virtual Herbarium |
| 23 | *Metasequoia glyptostroboides* Hu & W. C. Cheng | 105.3112 | 28.1613 | Chinese Virtual Herbarium |
| 24 | *Metasequoia glyptostroboides* Hu & W. C. Cheng | 105.4532 | 26.422 | Chinese Virtual Herbarium |
| 25 | *Metasequoia glyptostroboides* Hu & W. C. Cheng | 108.43459 | 27.53189 | Chinese Virtual Herbarium |
| 26 | *Metasequoia glyptostroboides* Hu & W. C. Cheng | 114.3023 | 28.2119 | Chinese Virtual Herbarium |
| 27 | *Metasequoia glyptostroboides* Hu & W. C. Cheng | 114.35 | 28.332 | Chinese Virtual Herbarium |
| 28 | *Metasequoia glyptostroboides* Hu & W. C. Cheng | 114.221 | 28.3002 | Chinese Virtual Herbarium |
| 29 | *Metasequoia glyptostroboides* Hu & W. C. Cheng | 114.1715 | 27.4441 | Chinese Virtual Herbarium |
| 30 | *Metasequoia glyptostroboides* Hu & W. C. Cheng | 107.3559 | 30.2414 | Chinese Virtual Herbarium |
| 31 | *Metasequoia glyptostroboides* Hu & W. C. Cheng | 108.431661 | 31.171807 | Chinese Virtual Herbarium |
| 32 | *Metasequoia glyptostroboides* Hu & W. C. Cheng | 107.624 | 30.512 | Chinese Virtual Herbarium |
| 33 | *Metasequoia glyptostroboides* Hu & W. C. Cheng | 108.474688 | 29.335323 | Chinese Virtual Herbarium |
| 34 | *Metasequoia glyptostroboides* Hu & W. C. Cheng | 107.6157344 | 27.70065351 | Chinese Virtual Herbarium |
| 35 | *Metasequoia glyptostroboides* Hu & W. C. Cheng | 106.421729 | 28.66467239 | Chinese Virtual Herbarium |
| 36 | *Metasequoia glyptostroboides* Hu & W. C. Cheng | 116.342574 | 39.91649408 | Chinese Virtual Herbarium |
| 37 | *Metasequoia glyptostroboides* Hu & W. C. Cheng | 118.2475501 | 25.49730859 | Chinese Virtual Herbarium |
| 38 | *Metasequoia glyptostroboides* Hu & W. C. Cheng | 113.6285571 | 35.73110307 | Chinese Virtual Herbarium |
| 39 | *Metasequoia glyptostroboides* Hu & W. C. Cheng | 116.0514259 | 29.45453917 | Chinese Virtual Herbarium |
| 40 | *Metasequoia glyptostroboides* Hu & W. C. Cheng | 115.9330151 | 29.52070974 | Chinese Virtual Herbarium |
| 41 | *Metasequoia glyptostroboides* Hu & W. C. Cheng | 116.4854685 | 29.68890363 | Chinese Virtual Herbarium |
| 42 | *Metasequoia glyptostroboides* Hu & W. C. Cheng | 116.342574 | 39.91649408 | Chinese Virtual Herbarium |
| 43 | *Metasequoia glyptostroboides* Hu & W. C. Cheng | 114.5094739 | 36.86112734 | Chinese Virtual Herbarium |
| 44 | *Metasequoia glyptostroboides* Hu & W. C. Cheng | 106.6818479 | 26.44429703 | Chinese Virtual Herbarium |
| 45 | *Metasequoia glyptostroboides* Hu & W. C. Cheng | 106.5584342 | 29.56899625 | Chinese Virtual Herbarium |
| 46 | *Metasequoia glyptostroboides* Hu & W. C. Cheng | 120.4406017 | 37.36113714 | Chinese Virtual Herbarium |
| 47 | *Metasequoia glyptostroboides* Hu & W. C. Cheng | 118.8376791 | 32.06043369 | Chinese Virtual Herbarium |
| 48 | *Metasequoia glyptostroboides* Hu & W. C. Cheng | 121.3886119 | 31.11884258 | Chinese Virtual Herbarium |
| 49 | *Metasequoia glyptostroboides* Hu & W. C. Cheng | 121.34848 | 30.74785238 | Chinese Virtual Herbarium |
| 50 | *Metasequoia glyptostroboides* Hu & W. C. Cheng | 121.349645 | 31.28956788 | Chinese Virtual Herbarium |
| 51 | *Metasequoia glyptostroboides* Hu & W. C. Cheng | 121.7140182 | 31.1067206 | Chinese Virtual Herbarium |
| 52 | *Metasequoia glyptostroboides* Hu & W. C. Cheng | 121.4805037 | 30.92372011 | Chinese Virtual Herbarium |
| 53 | *Metasequoia glyptostroboides* Hu & W. C. Cheng | 106.2004367 | 32.65675023 | Chinese Virtual Herbarium |
| 54 | *Metasequoia glyptostroboides* Hu & W. C. Cheng | 118.5471512 | 36.18924641 | Chinese Virtual Herbarium |
| 55 | *Metasequoia glyptostroboides* Hu & W. C. Cheng | 118.4714915 | 35.55609767 | Chinese Virtual Herbarium |
| 56 | *Metasequoia glyptostroboides* Hu & W. C. Cheng | 121.4544154 | 37.47003838 | Chinese Virtual Herbarium |
| 57 | *Metasequoia glyptostroboides* Hu & W. C. Cheng | 118.0424631 | 35.44113772 | Chinese Virtual Herbarium |
| 58 | *Metasequoia glyptostroboides* Hu & W. C. Cheng | 114.3023 | 28.2119 | Chinese Virtual Herbarium |
| 59 | *Metasequoia glyptostroboides* Hu & W. C. Cheng | 114.35 | 28.332 | Chinese Virtual Herbarium |
| 60 | *Metasequoia glyptostroboides* Hu & W. C. Cheng | 107.3759 | 30.2414 | Chinese Virtual Herbarium |
| 61 | *Metasequoia glyptostroboides* Hu & W. C. Cheng | 108.431661 | 31.171807 | Chinese Virtual Herbarium |
| 62 | *Metasequoia glyptostroboides* Hu & W. C. Cheng | 107.624 | 30.512 | Chinese Virtual Herbarium |
| 63 | *Metasequoia glyptostroboides* Hu & W. C. Cheng | 108.474688 | 29.333523 | Chinese Virtual Herbarium |
| 64 | *Metasequoia glyptostroboides* Hu & W. C. Cheng | 113.669352 | 34.79221299 | Chinese Virtual Herbarium |
| 65 | *Metasequoia glyptostroboides* Hu & W. C. Cheng | 106.7274867 | 28.7458865 | Chinese Virtual Herbarium |
| 66 | *Metasequoia glyptostroboides* Hu & W. C. Cheng | 114.117002 | 22.54581297 | Chinese Virtual Herbarium |
| 67 | *Metasequoia glyptostroboides* Hu & W. C. Cheng | 107.5511095 | 29.39989762 | Chinese Virtual Herbarium |
| 68 | *Metasequoia glyptostroboides* Hu & W. C. Cheng | 106.421729 | 28.66467239 | Chinese Virtual Herbarium |
| 69 | *Metasequoia glyptostroboides* Hu & W. C. Cheng | 115.8154653 | 29.45077294 | Chinese Virtual Herbarium |
| 70 | *Metasequoia glyptostroboides* Hu & W. C. Cheng | 116.1076036 | 39.94614672 | Chinese Virtual Herbarium |
| 71 | *Metasequoia glyptostroboides* Hu & W. C. Cheng | 113.841162 | 31.62211428 | Chinese Virtual Herbarium |
| 72 | *Metasequoia glyptostroboides* Hu & W. C. Cheng | 111.9341906 | 33.50549 | Chinese Virtual Herbarium |
| 73 | *Metasequoia glyptostroboides* Hu & W. C. Cheng | 110.3183269 | 27.27836365 | Chinese Virtual Herbarium |
| 74 | *Metasequoia glyptostroboides* Hu & W. C. Cheng | 107.1055851 | 29.1634786 | Chinese Virtual Herbarium |
| 75 | *Metasequoia glyptostroboides* Hu & W. C. Cheng | 112.7498022 | 27.23715453 | Chinese Virtual Herbarium |
| 76 | *Metasequoia glyptostroboides* Hu & W. C. Cheng | 107.6194838 | 28.86864981 | Chinese Virtual Herbarium |
| 77 | *Metasequoia glyptostroboides* Hu & W. C. Cheng | 121.7579461 | 37.26526972 | Chinese Virtual Herbarium |
| 78 | *Metasequoia glyptostroboides* Hu & W. C. Cheng | 106.7144759 | 26.60402954 | Chinese Virtual Herbarium |
| 79 | *Metasequoia glyptostroboides* Hu & W. C. Cheng | 107.7760973 | 30.66055326 | Chinese Virtual Herbarium |
| 80 | *Metasequoia glyptostroboides* Hu & W. C. Cheng | 108.6716116 | 31.95339075 | Chinese Virtual Herbarium |
| 81 | *Metasequoia glyptostroboides* Hu & W. C. Cheng | 108.1204142 | 30.0061087 | China Knowledge Network |
| 82 | *Metasequoia glyptostroboides* Hu & W. C. Cheng | 109.4504157 | 29.46412177 | China Knowledge Network |
| 83 | *Metasequoia glyptostroboides* Hu & W. C. Cheng | 108.7326904 | 30.0243635 | China Knowledge Network |
| 84 | *Metasequoia glyptostroboides* Hu & W. C. Cheng | 108.9424806 | 30.29720344 | China Knowledge Network |
| 85 | *Metasequoia glyptostroboides* Hu & W. C. Cheng | 108.6913904 | 30.44102971 | China Knowledge Network |
| 86 | *Metasequoia glyptostroboides* Hu & W. C. Cheng | 108.5444463 | 30.44152244 | China Knowledge Network |
| 87 | *Metasequoia glyptostroboides* Hu & W. C. Cheng | 108.9424806 | 30.29720344 | China Knowledge Network |
| 88 | *Metasequoia glyptostroboides* Hu & W. C. Cheng | 117.5385854 | 32.88097673 | China Knowledge Network |
| 89 | *Metasequoia glyptostroboides* Hu & W. C. Cheng | 108.61 | 30.09 | DOI： 10.13292/j.1000-4890.201906.018 |
| 90 | *Metasequoia glyptostroboides* Hu & W. C. Cheng | 108.62 | 30.15 | DOI： 10.13292/j.1000-4890.201906.018 |
| 91 | *Metasequoia glyptostroboides* Hu & W. C. Cheng | 108.8 | 29.97 | DOI： 10.13292/j.1000-4890.201906.018 |
| 92 | *Metasequoia glyptostroboides* Hu & W. C. Cheng | 108.72 | 30.05 | DOI： 10.13292/j.1000-4890.201906.018 |
| 93 | *Metasequoia glyptostroboides* Hu & W. C. Cheng | 108.67 | 30.12 | DOI： 10.13292/j.1000-4890.201906.018 |
| 94 | *Metasequoia glyptostroboides* Hu & W. C. Cheng | 108.7 | 30.07 | DOI： 10.13292/j.1000-4890.201906.018 |
| 95 | *Metasequoia glyptostroboides* Hu & W. C. Cheng | 108.63 | 30.06 | DOI： 10.13292/j.1000-4890.201906.018 |
| 96 | *Metasequoia glyptostroboides* Hu & W. C. Cheng | 108.71 | 29.97 | DOI： 10.13292/j.1000-4890.201906.018 |
| 97 | *Metasequoia glyptostroboides* Hu & W. C. Cheng | 109.12 | 29.76 | DOI： 10.13292/j.1000-4890.201906.018 |
| 98 | *Metasequoia glyptostroboides* Hu & W. C. Cheng | 108.57 | 30.17 | DOI： 10.13292/j.1000-4890.201906.018 |
| 99 | *Metasequoia glyptostroboides* Hu & W. C. Cheng | 108.70 | 30.44 | DOI： 10.13292/j.1000-4890.201906.018 |
| 100 | *Metasequoia glyptostroboides* Hu & W. C. Cheng | 108.59 | 30.38 | DOI： 10.13292/j.1000-4890.201906.018 |
| 101 | *Metasequoia glyptostroboides* Hu & W. C. Cheng | 108.78 | 30.22 | DOI： 10.13292/j.1000-4890.201906.018 |
| 102 | *Metasequoia glyptostroboides* Hu & W. C. Cheng | 10.53 | 29.21 | DOI： 10.13292/j.1000-4890.201906.018 |
| 103 | *Metasequoia glyptostroboides* Hu & W. C. Cheng | 108.47 | 30.21 | DOI： 10.13292/j.1000-4890.201906.018 |
| 104 | *Glyptostrobus pensilis* (Staunton ex D. Don) K. Koch | 116.0334207 | 25.89166627 | Chinese Virtual Herbarium |
| 105 | *Glyptostrobus pensilis* (Staunton ex D. Don) K. Koch | 109.2992641 | 34.64301106 | Chinese Virtual Herbarium |
| 106 | *Glyptostrobus pensilis* (Staunton ex D. Don) K. Koch | 113.0667958 | 22.52951456 | Chinese Virtual Herbarium |
| 107 | *Glyptostrobus pensilis* (Staunton ex D. Don) K. Koch | 115.0365817 | 37.06909639 | Chinese Virtual Herbarium |
| 108 | *Glyptostrobus pensilis* (Staunton ex D. Don) K. Koch | 120.1246406 | 30.2597771 | Chinese Virtual Herbarium |
| 109 | *Glyptostrobus pensilis* (Staunton ex D. Don) K. Koch | 110.8634907 | 26.4397237 | Chinese Virtual Herbarium |
| 110 | *Glyptostrobus pensilis* (Staunton ex D. Don) K. Koch | 113.3737806 | 23.18794026 | Chinese Virtual Herbarium |
| 111 | *Glyptostrobus pensilis* (Staunton ex D. Don) K. Koch | 115.8154653 | 29.45077294 | Chinese Virtual Herbarium |
| 112 | *Glyptostrobus pensilis* (Staunton ex D. Don) K. Koch | 110.193241 | 25.28499188 | Chinese Virtual Herbarium |
| 113 | *Glyptostrobus pensilis* (Staunton ex D. Don) K. Koch | 113.3004534 | 22.81045343 | Chinese Virtual Herbarium |
| 114 | *Glyptostrobus pensilis* (Staunton ex D. Don) K. Koch | 117.9494596 | 28.46062592 | Chinese Virtual Herbarium |
| 115 | *Glyptostrobus pensilis* (Staunton ex D. Don) K. Koch | 115.8154653 | 29.45077294 | Chinese Virtual Herbarium |
| 116 | *Glyptostrobus pensilis* (Staunton ex D. Don) K. Koch | 118.562276 | 26.03971596 | Chinese Virtual Herbarium |
| 117 | *Glyptostrobus pensilis* (Staunton ex D. Don) K. Koch | 110.327247 | 25.03963086 | Chinese Virtual Herbarium |
| 118 | *Glyptostrobus pensilis* (Staunton ex D. Don) K. Koch | 108.656226 | 23.17215196 | Chinese Virtual Herbarium |
| 119 | *Glyptostrobus pensilis* (Staunton ex D. Don) K. Koch | 116.9984919 | 28.12075556 | Chinese Virtual Herbarium |
| 120 | *Glyptostrobus pensilis* (Staunton ex D. Don) K. Koch | 121.4509489 | 31.15387392 | Chinese Virtual Herbarium |
| 121 | *Glyptostrobus pensilis* (Staunton ex D. Don) K. Koch | 121.189029 | 31.08165586 | Chinese Virtual Herbarium |
| 122 | *Glyptostrobus pensilis* (Staunton ex D. Don) K. Koch | 101.262951 | 21.93160414 | Chinese Virtual Herbarium |
| 123 | *Glyptostrobus pensilis* (Staunton ex D. Don) K. Koch | 119.4123794 | 26.76518066 | Chinese Virtual Herbarium |
| 124 | *Glyptostrobus pensilis* (Staunton ex D. Don) K. Koch | 108.300378 | 22.84912699 | Chinese Virtual Herbarium |
| 125 | *Glyptostrobus pensilis* (Staunton ex D. Don) K. Koch | 113.5210997 | 26.21329912 | Chinese Virtual Herbarium |
| 126 | *Glyptostrobus pensilis* (Staunton ex D. Don) K. Koch | 114.12688 | 22.57220398 | Chinese Virtual Herbarium |
| 127 | *Glyptostrobus pensilis* (Staunton ex D. Don) K. Koch | 115.9606612 | 29.66666085 | Chinese Virtual Herbarium |
| 128 | *Glyptostrobus pensilis* (Staunton ex D. Don) K. Koch | 110.8634907 | 26.4397237 | Chinese Virtual Herbarium |
| 129 | *Glyptostrobus pensilis* (Staunton ex D. Don) K. Koch | 117.6888636 | 27.74830895 | Chinese Virtual Herbarium |
| 130 | *Glyptostrobus pensilis* (Staunton ex D. Don) K. Koch | 118.2795705 | 30.1781607 | Chinese Virtual Herbarium |
| 131 | *Glyptostrobus pensilis* (Staunton ex D. Don) K. Koch | 114.2955559 | 26.75370974 | Chinese Virtual Herbarium |
| 132 | *Glyptostrobus pensilis* (Staunton ex D. Don) K. Koch | 109.143567 | 26.23687405 | Chinese Virtual Herbarium |
| 133 | *Glyptostrobus pensilis* (Staunton ex D. Don) K. Koch | 109.180508 | 18.75587149 | Chinese Virtual Herbarium |
| 134 | *Glyptostrobus pensilis* (Staunton ex D. Don) K. Koch | 117.718856 | 26.71672344 | Chinese Virtual Herbarium |
| 135 | *Glyptostrobus pensilis* (Staunton ex D. Don) K. Koch | 118.8291433 | 32.08482347 | Chinese Virtual Herbarium |
| 136 | *Glyptostrobus pensilis* (Staunton ex D. Don) K. Koch | 114.075154 | 32.13705504 | Chinese Virtual Herbarium |
| 137 | *Glyptostrobus pensilis* (Staunton ex D. Don) K. Koch | 114.363708 | 30.4817799 | Chinese Virtual Herbarium |
| 138 | *Glyptostrobus pensilis* (Staunton ex D. Don) K. Koch | 118.087351 | 27.38910499 | Chinese Virtual Herbarium |
| 139 | *Glyptostrobus pensilis* (Staunton ex D. Don) K. Koch | 102.7585262 | 25.12237228 | Chinese Virtual Herbarium |
| 140 | *Glyptostrobus pensilis* (Staunton ex D. Don) K. Koch | 119.5745534 | 30.10387155 | Chinese Virtual Herbarium |
| 141 | *Glyptostrobus pensilis* (Staunton ex D. Don) K. Koch | 120.2260967 | 31.54481111 | Chinese Virtual Herbarium |
| 142 | *Glyptostrobus pensilis* (Staunton ex D. Don) K. Koch | 114.5585486 | 25.7905188 | Chinese Virtual Herbarium |
| 143 | *Glyptostrobus pensilis* (Staunton ex D. Don) K. Koch | 113.343837 | 25.114857 | Chinese Virtual Herbarium |
| 144 | *Glyptostrobus pensilis* (Staunton ex D. Don) K. Koch | 116.005616 | 29.301223 | Chinese Virtual Herbarium |
| 145 | *Glyptostrobus pensilis* (Staunton ex D. Don) K. Koch | 110.202902 | 25.191779 | Chinese Virtual Herbarium |
| 146 | *Glyptostrobus pensilis* (Staunton ex D. Don) K. Koch | 118.083 | 24.45 | Chinese Virtual Herbarium |
| 147 | *Glyptostrobus pensilis* (Staunton ex D. Don) K. Koch | 110.131865 | 25.044637 | Chinese Virtual Herbarium |
| 148 | *Glyptostrobus pensilis* (Staunton ex D. Don) K. Koch | 114.3646 | 27.4421 | Chinese Virtual Herbarium |
| 149 | *Glyptostrobus pensilis* (Staunton ex D. Don) K. Koch | 114.3 | 22.4 | GBIF |
| 150 | *Glyptostrobus pensilis* (Staunton ex D. Don) K. Koch | 118.7 | 32.4 | GBIF |
| 151 | *Glyptostrobus pensilis* (Staunton ex D. Don) K. Koch | 114.3 | 22.4 | GBIF |
| 152 | *Glyptostrobus pensilis* (Staunton ex D. Don) K. Koch | 118.5531773 | 26.07850628 | China Knowledge Network |
| 153 | *Glyptostrobus pensilis* (Staunton ex D. Don) K. Koch | 118.9925962 | 26.91426492 | China Knowledge Network |
| 154 | *Glyptostrobus pensilis* (Staunton ex D. Don) K. Koch | 109.8713574 | 25.73202355 | China Knowledge Network |
| 155 | *Glyptostrobus pensilis* (Staunton ex D. Don) K. Koch | 118.2475501 | 25.49730859 | China Knowledge Network |
| 156 | *Glyptostrobus pensilis* (Staunton ex D. Don) K. Koch | 113.3034673 | 22.21551566 | China Knowledge Network |
| 157 | *Glyptostrobus pensilis* (Staunton ex D. Don) K. Koch | 113.7672638 | 25.13814277 | China Knowledge Network |
| 158 | *Glyptostrobus pensilis* (Staunton ex D. Don) K. Koch | 118.1001585 | 26.11003485 | China Knowledge Network |
| 159 | *Glyptostrobus pensilis* (Staunton ex D. Don) K. Koch | 118.8691708 | 27.01657707 | China Knowledge Network |
| 160 | *Glyptostrobus pensilis* (Staunton ex D. Don) K. Koch | 117.3543523 | 28.42134962 | China Knowledge Network |
| 161 | *Glyptostrobus pensilis* (Staunton ex D. Don) K. Koch | 118.1239192 | 26.57637856 | China Knowledge Network |
| 162 | *Glyptostrobus pensilis* (Staunton ex D. Don) K. Koch | 113.2147498 | 22.32091215 | China Knowledge Network |
| 163 | *Glyptostrobus pensilis* (Staunton ex D. Don) K. Koch | 119.2926919 | 26.16787932 | China Knowledge Network |
| 164 | *Glyptostrobus pensilis* (Staunton ex D. Don) K. Koch | 114.6085369 | 27.44959326 | China Knowledge Network |
| 165 | *Glyptostrobus pensilis* (Staunton ex D. Don) K. Koch | 113.3443609 | 22.26102315 | China Knowledge Network |
| 166 | *Glyptostrobus pensilis* (Staunton ex D. Don) K. Koch | 113.643794 | 24.65225498 | China Knowledge Network |
| 167 | *Glyptostrobus pensilis* (Staunton ex D. Don) K. Koch | 116.0514259 | 29.45453917 | China Knowledge Network |
| 168 | *Glyptostrobus pensilis* (Staunton ex D. Don) K. Koch | 109.4995593 | 30.28788793 | China Knowledge Network |
| 169 | *Glyptostrobus pensilis* (Staunton ex D. Don) K. Koch | 119.4162 | 25.72617 | Ye et al.,2021. https://doi.org/10.3390/f13020257 |
| 170 | *Glyptostrobus pensilis* (Staunton ex D. Don) K. Koch | 119.325 | 27.19945 | Ye et al.,2021. https://doi.org/10.3390/f13020257 |
| 171 | *Glyptostrobus pensilis* (Staunton ex D. Don) K. Koch | 119.15 | 26.68904 | Ye et al.,2021. https://doi.org/10.3390/f13020257 |
| 172 | *Glyptostrobus pensilis* (Staunton ex D. Don) K. Koch | 119.0353 | 26.89387 | Ye et al.,2021. https://doi.org/10.3390/f13020257 |
| 173 | *Glyptostrobus pensilis* (Staunton ex D. Don) K. Koch | 118.8623 | 27.00615 | Ye et al.,2021. https://doi.org/10.3390/f13020257 |
| 174 | *Glyptostrobus pensilis* (Staunton ex D. Don) K. Koch | 118.8158 | 25.55034 | Ye et al.,2021. https://doi.org/10.3390/f13020257 |
| 175 | *Glyptostrobus pensilis* (Staunton ex D. Don) K. Koch | 118.5293 | 26.05559 | Ye et al.,2021. https://doi.org/10.3390/f13020257 |
| 176 | *Glyptostrobus pensilis* (Staunton ex D. Don) K. Koch | 118.4092 | 25.76508 | Ye et al.,2021. https://doi.org/10.3390/f13020257 |
| 177 | *Glyptostrobus pensilis* (Staunton ex D. Don) K. Koch | 118.3262 | 26.08217 | Ye et al.,2021. https://doi.org/10.3390/f13020257 |
| 178 | *Glyptostrobus pensilis* (Staunton ex D. Don) K. Koch | 117.7625 | 28.35442 | Ye et al.,2021. https://doi.org/10.3390/f13020257 |
| 179 | *Glyptostrobus pensilis* (Staunton ex D. Don) K. Koch | 117.5252 | 28.49783 | Ye et al.,2021. https://doi.org/10.3390/f13020257 |
| 180 | *Glyptostrobus pensilis* (Staunton ex D. Don) K. Koch | 117.3524 | 28.35964 | Ye et al.,2021. https://doi.org/10.3390/f13020257 |
| 181 | *Glyptostrobus pensilis* (Staunton ex D. Don) K. Koch | 117.2628 | 28.2192 | Ye et al.,2021. https://doi.org/10.3390/f13020257 |
| 182 | *Glyptostrobus pensilis* (Staunton ex D. Don) K. Koch | 116.9694 | 28.2807 | Ye et al.,2021. https://doi.org/10.3390/f13020257 |
| 183 | *Glyptostrobus pensilis* (Staunton ex D. Don) K. Koch | 113.7004 | 23.58032 | Ye et al.,2021. https://doi.org/10.3390/f13020257 |
| 184 | *Glyptostrobus pensilis* (Staunton ex D. Don) K. Koch | 113.5373 | 26.21137 | Ye et al.,2021. https://doi.org/10.3390/f13020257 |
| 185 | *Glyptostrobus pensilis* (Staunton ex D. Don) K. Koch | 113.2591 | 22.39246 | Ye et al.,2021. https://doi.org/10.3390/f13020257 |
| 186 | *Glyptostrobus pensilis* (Staunton ex D. Don) K. Koch | 112.3101 | 24.3201 | Ye et al.,2021. https://doi.org/10.3390/f13020257 |
| 187 | *Glyptostrobus pensilis* (Staunton ex D. Don) K. Koch | 111.0501 | 22.01009 | Ye et al.,2021. https://doi.org/10.3390/f13020257 |
| 188 | *Glyptostrobus pensilis* (Staunton ex D. Don) K. Koch | 108.2839 | 12.98667 | Ye et al.,2021. https://doi.org/10.3390/f13020257 |
| 189 | *Glyptostrobus pensilis* (Staunton ex D. Don) K. Koch | 108.2825 | 12.93528 | Ye et al.,2021. https://doi.org/10.3390/f13020257 |
| 190 | *Glyptostrobus pensilis* (Staunton ex D. Don) K. Koch | 108.2811 | 13.22944 | Ye et al.,2021. https://doi.org/10.3390/f13020257 |
| 191 | *Glyptostrobus pensilis* (Staunton ex D. Don) K. Koch | 105.5364 | 17.90028 | Ye et al.,2021. https://doi.org/10.3390/f13020257 |
| 192 | *Glyptostrobus pensilis* (Staunton ex D. Don) K. Koch | 105.4833 | 17.88333 | Ye et al.,2021. https://doi.org/10.3390/f13020257 |
| 193 | *Glyptostrobus pensilis* (Staunton ex D. Don) K. Koch | 105.2378 | 18.02694 | Ye et al.,2021. https://doi.org/10.3390/f13020257 |
| 194 | *Glyptostrobus pensilis* (Staunton ex D. Don) K. Koch | 105.2333 | 18.01667 | Ye et al.,2021. https://doi.org/10.3390/f13020257 |
| 195 | *Glyptostrobus pensilis* (Staunton ex D. Don) K. Koch | 119.3419 | 26.49986 | Ye et al.,2021. https://doi.org/10.3390/f13020257 |
| 196 | *Thuja sutchuenensis* Franch*.* | 105.4 | 32.1 | GBIF |
| 197 | *Thuja sutchuenensis* Franch*.* | 108.8 | 31.9 | GBIF |
| 198 | *Thuja sutchuenensis* Franch*.* | 108.4021 | 31.3659 | Chinese Virtual Herbarium |
| 199 | *Thuja sutchuenensis* Franch*.* | 100.17437 | 29.57567 | Chinese Virtual Herbarium |
| 200 | *Thuja sutchuenensis* Franch*.* | 100.024884 | 28.08384 | Chinese Virtual Herbarium |
| 201 | *Thuja sutchuenensis* Franch*.* | 108.45314 | 31.42561 | Chinese Virtual Herbarium |
| 202 | *Thuja sutchuenensis* Franch*.* | 108.820697 | 31.73816357 | Chinese Virtual Herbarium |
| 203 | *Thuja sutchuenensis* Franch*.* | 108.7946057 | 31.59857733 | Chinese Virtual Herbarium |
| 204 | *Thuja sutchuenensis* Franch*.* | 108.7353262 | 31.66547418 | Chinese Virtual Herbarium |
| 205 | *Thuja sutchuenensis* Franch*.* | 108.6716116 | 31.95339075 | Chinese Virtual Herbarium |
| 206 | *Thuja sutchuenensis* Franch*.* | 109.0158199 | 31.81798551 | China Knowledge Network |
| 207 | *Thuja sutchuenensis* Franch*.* | 108.3994977 | 31.16664411 | China Knowledge Network |
| 208 | *Thuja sutchuenensis* Franch*.* | 108.45 | 31.42 | China Knowledge Network |
| 209 | *Thuja sutchuenensis* Franch*.* | 108.4 | 31.36 | China Knowledge Network |
| 210 | *Thuja sutchuenensis* Franch*.* | 108.75 | 31.71 | Tao et al.https://doi.org/10.1016/j.pld.2023.06.005 |
| 211 | *Thuja sutchuenensis* Franch*.* | 108.72 | 31.66 | Tao et al.https://doi.org/10.1016/j.pld.2023.06.005 |
| 212 | *Thuja sutchuenensis* Franch*.* | 108.83 | 31.6 | Tao et al.https://doi.org/10.1016/j.pld.2023.06.005 |
| 213 | *Thuja sutchuenensis* Franch*.* | 108.68 | 31.62 | Tao et al.https://doi.org/10.1016/j.pld.2023.06.005 |
| 214 | *Thuja sutchuenensis* Franch*.* | 108.43 | 31.63 | Tao et al.https://doi.org/10.1016/j.pld.2023.06.005 |
| 215 | *Thuja sutchuenensis* Franch*.* | 108.844 | 31.598 | Qin et al.https://doi.org/10.1016/j.gecco.2020.e01430 |
| 216 | *Thuja sutchuenensis* Franch*.* | 108.666 | 31.606 | Qin et al.https://doi.org/10.1016/j.gecco.2020.e01430 |
| 217 | *Thuja sutchuenensis* Franch*.* | 108.668 | 31.611 | Qin et al.https://doi.org/10.1016/j.gecco.2020.e01430 |
| 218 | *Thuja sutchuenensis* Franch*.* | 108.698 | 31.656 | Qin et al.https://doi.org/10.1016/j.gecco.2020.e01430 |
| 219 | *Thuja sutchuenensis* Franch*.* | 108.78 | 31.721 | Qin et al.https://doi.org/10.1016/j.gecco.2020.e01430 |
| 220 | *Thuja sutchuenensis* Franch*.* | 108.413 | 31.638 | Qin et al.https://doi.org/10.1016/j.gecco.2020.e01430 |

Table S2: Environmental factors involved in modeling.

| Species | Type | Variable code |
| --- | --- | --- |
| *Metasequoia glyptostroboides* Hu & W. C. Cheng | Climatic factor | bio3 |
|  |  | bio5 |
|  |  | bio6 |
|  |  | bio8 |
|  |  | bio13 |
|  |  | bio14 |
|  |  | bio15 |
|  |  | bio18 |
|  | Topographic factor | elev |
|  | Soil factor | t_esp |
|  |  | t_oc |
|  |  | t_bs |
|  |  | t_silt |
| *Glyptostrobus pensilis* (Staunton ex D. Don) K. Koch | Climatic factor | bio2 |
|  |  | bio3 |
|  |  | bio5 |
|  |  | bio6 |
|  |  | bio8 |
|  |  | bio14 |
|  |  | bio15 |
|  |  | bio18 |
|  | Topographic factor | elev |
|  | Soil factor | t_grave |
|  |  | t_oc |
|  |  | t_bs |
|  |  | t_silt |
| *Thuja sutchuenensis* Franch*.* | Climatic factor | bio5 |
|  |  | bio6 |
|  |  | bio7 |
|  |  | bio15 |
|  |  | bio18 |
|  | Topographic factor | elev |
|  | Soil factor | t_ph_h2o |
|  |  | t_silt |


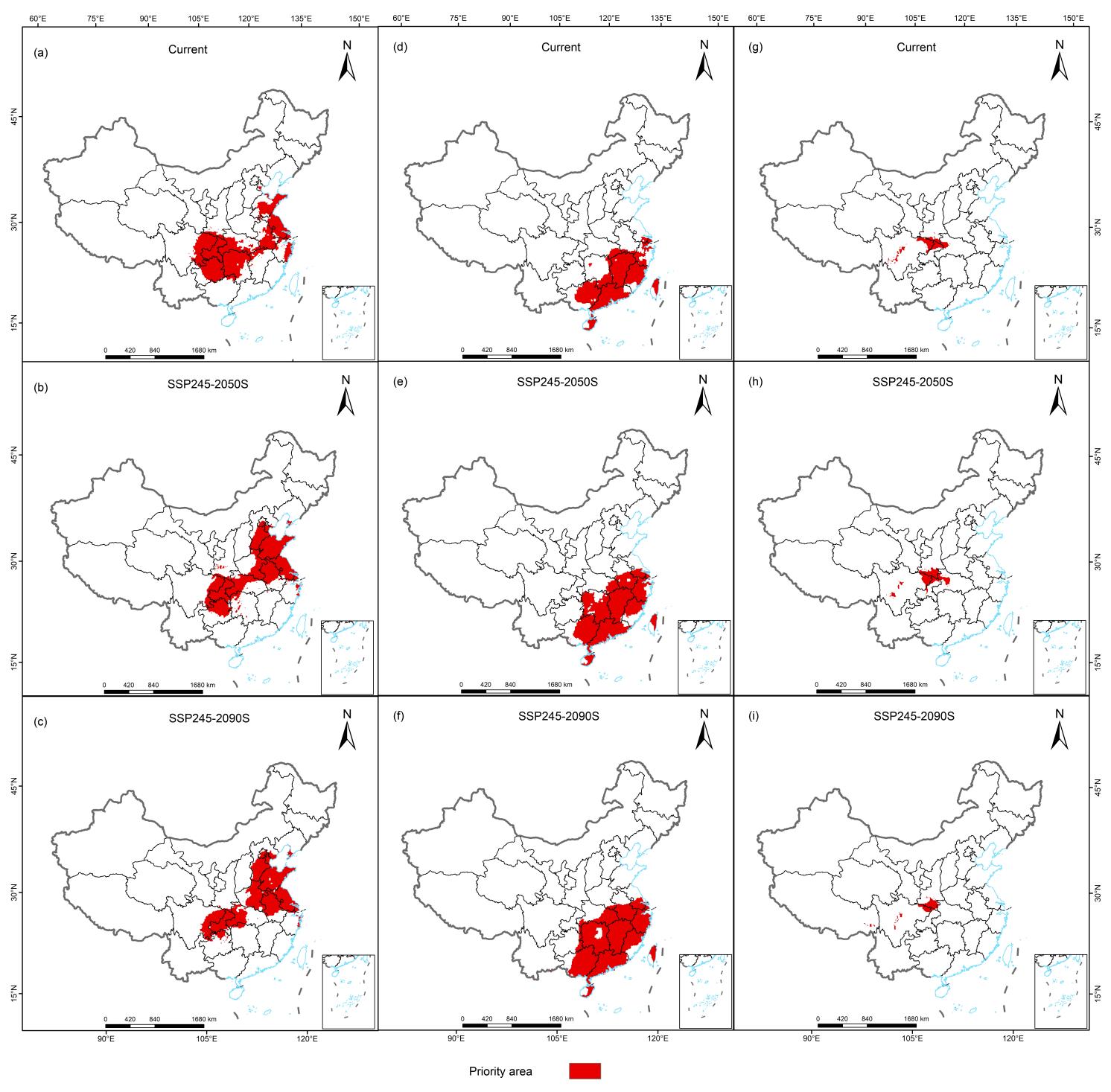


Figure S1. Priority conservation area plan for three Cupressaceae species: (a)-(c) are *Metasequoia glyptostroboides* Hu & W. C. Cheng, (d)-(f) are *Glyptostrobus pensilis* (Staunton ex D. Don) K. Koch, and (g)-(i) are *Thuja sutchuenensis Franch*.
